# Tumor Cell Plasticity in Cancer: Signaling Pathways and Pharmaceutical Interventions

**DOI:** 10.1002/mco2.70541

**Published:** 2025-12-17

**Authors:** Shangwei Sun, Yunwei Sun, Ling Lan, Siyuan Luan, Jin Zhou, Jiehui Deng, Yong Yuan, Zhong Wu

**Affiliations:** ^1^ Department of Thoracic Surgery West China Hospital, Sichuan University Chengdu Sichuan China; ^2^ Division of Pancreatic Surgery, Department of General Surgery West China Hospital, Sichuan University Chengdu Sichuan China; ^3^ Department of Biotherapy, Cancer Center and State Key Laboratory of Biotherapy West China Hospital of Sichuan University Chengdu Sichuan China; ^4^ Division of Liver Surgery, Department of General Surgery West China Hospital, Sichuan University Chengdu Sichuan China; ^5^ Laura and Isaac Perlmutter Cancer Center New York University Grossman School of Medicine New York New York USA

**Keywords:** differentiation therapy, epigenetic reprogramming, spatiotemporal omics, therapy resistance, tumor cell plasticity, tumor evolution, tumor microenvironment

## Abstract

Cellular plasticity, the ability of cells to dynamically alter their phenotypes, is a key driver of tumor evolution. This process is a hallmark of cancer which enables the acquisition of malignant traits, leading to metastasis, progression, and therapy resistance. It is governed by cell‐intrinsic factors, such as genomic instability and epigenetic reprogramming, and extrinsic stimuli from the tumor microenvironment. However, a unified framework is still needed to position plasticity as the central process that links these drivers to diverse cancer hallmarks. In this review, we first explore how plasticity enables key steps of tumor evolution, including tumorigenesis, metastasis driven by epithelial–mesenchymal plasticity (EMP), therapy resistance, and cancer stem cell (CSC) dynamics. We then summarize the intrinsic and extrinsic mechanisms that govern this adaptability. Finally, we discuss clinical advances in monitoring and targeting plasticity and highlight how new spatiotemporal technologies can address current research challenges. This review provides a framework positioning cellular plasticity as a central mechanism in cancer evolution, connecting its fundamental drivers to clinical translation. By synthesizing the latest advances, we offer perspectives for developing therapies that integrate prediction, monitoring, and targeting of plasticity to proactively guide cancer evolution toward manageable outcomes.

## Graphical abstract

Cell plasticity refers to a dynamic program through which cells reprogram their phenotype, acquiring new histologic and molecular characteristics. Recognized as a hallmark of cancer, cellular plasticity plays a critical role in tumor evolution. It is driven by factors such as cancer cell‐intrinsic genomic instability, epigenetic reprogramming, transcriptional remodeling, and interactions with the tumor microenvironment. These factors enable tumor cells to acquire new traits, contributing to other hallmark features of cancer, including metastasis, progression, and resistance to therapy. As an essential intermediate state, cellular plasticity enables characteristics essential for oncogenic features, and offers a novel perspective on tumor evolution and adaptation during cancer progression and response to drug treatment. In this review, we explore the interactions between cancer cell plasticity and other hallmarks of cancer, and provide insights into tumor evolution in response to plasticity. We also highlight recent advances using novel spatiotemporal transcriptomics, which present promising technologies for future research into the mechanisms underlying cancer cell plasticity.

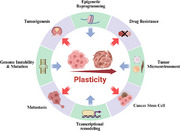

## 1 Introduction

Cellular plasticity is the ability of a cell to reprogram its phenotype and acquire new histological and molecular characteristics in response to intrinsic or extrinsic stimuli [1]. Stem cells, for example, exhibit plasticity through their capacity for both self‐renewal and differentiation into new phenotypes. Traditionally, this process was viewed as a unidirectional cascade, in which stem cells give rise to progressively more specialized progeny that ultimately lose their self‐renewal capacity. However, mature differentiated cells are not always terminally committed and can alter their fate through dedifferentiation (reverting to a more primitive, stem‐like state) and transdifferentiation (converting directly from one mature phenotype to another) [2, 3]. The experimental foundation for these concepts is based on two seminal discoveries: in 1987, Davis et al. demonstrated that a single transcription factor (TF), MyoD, could transdifferentiate mammalian fibroblasts into myoblasts [4], and in 2006, Takahashi and Yamanaka showed that a core set of four TFs (OSKM) could reprogramme adult somatic cells to a pluripotent state [5]. These findings highlight the remarkable extent of cellular plasticity. This capacity—from the canonical differentiation of stem cells to the latent reprogramming potential of somatic cells—is now understood to be a fundamental physiological process that enables the restoration of tissue homeostasis in response to insults such as tissue damage, inflammation or senescence [6, 7].

Nevertheless, accumulating evidence suggests that unlocking phenotypic plasticity of tumor cells was a critical component of cancer progression and has been defined as a new hallmark of cancer [8]. Indeed, cell plasticity seems to be different from the normal hallmarks and enabling characteristics. It can be facilitated by the enabling characteristics including genetic events, epigenetic fluctuations including both chromatin structure rearrangement and DNA methylation changes, and cell‐extrinsic factors [9, 10, 11, 12, 13, 14]. Moreover, previous research demonstrated that cell plasticity could promote multiple aspects of cancer phenotypes, including tumor initiation, progression, metastasis and resistance to therapy [1, 15, 16, 17, 18]. In a macro perspective, the unlocking of phenotypic plasticity appears to play an intermediate role, linking enabling characteristics to other hallmarks of cancer. In addition, as the progression of single‐cell RNA sequencing (scRNA‐seq) and lineage‐tracing methods, recent studies discovered several specialized highly plastic cell states during tumorigenesis, malignant progression, metastasis and therapy resistance [18, 19, 20, 21, 22, 23, 24]. These researches revealed cellular and molecular mechanisms of cell plasticity during special tumor evolution process, respectively. Taken together, these findings indicate that at the phenotypic level, cellular plasticity can be a result of enabling characteristics and contributes to the acquisition of other hallmarks and at the cellular level, it could also serve as a critical intermediate state during cell state transitions.

By enabling cancer cells to acquire the aggressive phenotypes required for tumor progression, therapeutic resistance, and metastasis, cellular plasticity establishes a crucial dependency that represents a compelling therapeutic vulnerability. However, the intrinsic complexity and dynamic nature of cellular plasticity have created significant challenges for understanding its underlying mechanisms. This has hindered clinical progress in developing robust monitoring strategies, validating therapeutic targets and designing dedicated clinical trials [25, 26]. In this review, we examine the various roles of cellular plasticity in tumor evolution—from promoting therapeutic resistance to enabling metastatic dissemination. We then summarize the intrinsic and extrinsic networks that govern this adaptability, evaluate emerging strategies for its monitoring and pharmacological targeting, and review the current clinical trial landscape. Finally, we outline emerging technologies and propose future directions for the clinical translation of therapies targeting plasticity.

## 2 Cancer Cell Plasticity Enables Tumor Evolution

**FIGURE 1:**
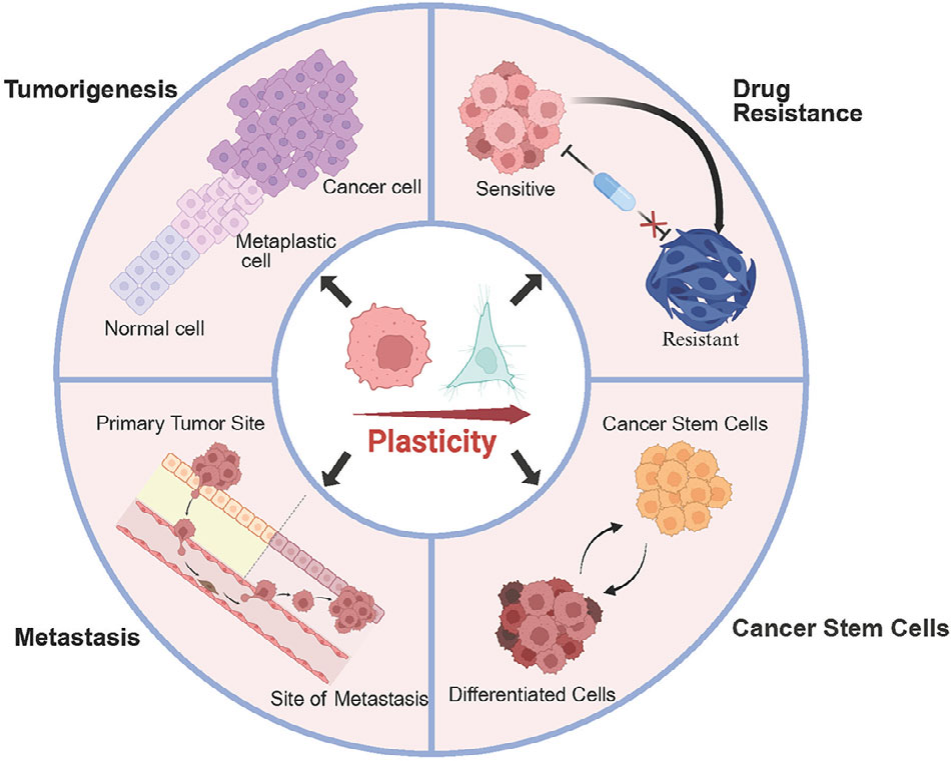
Cancer cell plasticity enables other cancer hallmarks. Throughout tumorigenesis, the metaplastic transformation of normal cells can represent an early, precancerous progression. Cancer cells may evade targeted therapies by undergoing lineage plasticity, transitioning from a therapy‐sensitive phenotype to a resistant one. During metastasis, epithelial–mesenchymal plasticity plays a crucial role, facilitating dissemination, colonization, and dormancy. The cancer stem cell (CSC) is increasingly viewed not as a rigidly defined cell population, but as a plastic cellular state that can be acquired by more differentiated tumor cells.

Cancer is a disease with great complexity and heterogeneity. Previous theory suggested that during cancer evolution, the normal cells undergo tumorigenesis, malignant progression, and ultimately acquire the distinctive hallmarks of cancer [8]. Cellular plasticity is a key driver of tumor evolution, enabling cancer cells to transition between distinct phenotypic states through transdifferentiation and dedifferentiation. This capacity is fundamental to acquiring malignant traits and overcoming diverse selective pressures. In this section, we introduce the essential roles of plasticity in the key processes of malignancy, including tumorigenesis, metastasis, and the development of adaptive resistance to therapy. Furthermore, we discuss how cellular plasticity impacts two fundamental concepts in oncology: the cancer stem cell (CSC) paradigm and intratumor heterogeneity (Figure 1).

### 2.1 Cancer Cell Plasticity in Tumorigenesis and Progression

Metaplasia represents a fundamental adaptive reprogramming event wherein a tissue responds to chronic injury or inflammation by replacing its native epithelium with a distinct, stress‐tolerant differentiated phenotype [27]. Metaplasia is believed to serve a protective role in response to chronic damage, either by replacing lost tissue or by forming barriers better adapted to withstand adverse conditions. However, in several organs, this phenomenon is associated with an increased susceptibility to cancer [3]. It is important to recognize that cell plasticity can drive these phenotypic shifts, as evidenced by acinar to ductal metaplasia (ADM) within the pancreas and breast [27]. In contrast, in Barrett's esophagus, where normal squamous epithelium is replaced by columnar cells resembling those of the small intestine, the original cell type responsible for intestinal metaplasia remains unidentified and may not be differentiated esophageal squamous cells [28]. In the following discussion, we will explore how cell plasticity contributes to tumorigenesis and progression, beginning with metaplasia.

#### 2.1.1 Acinar to Ductal Metaplasia in Pancreas

The transition of functional pancreatic acinar cells into a duct‐like state is defined as ADM. In both mouse models and human subjects, this plasticity mechanism is essential for the regeneration of acinar structures and pancreatic repopulation during chronic injury, such as pancreatitis [29]. While ADM lesions typically regress upon the resolution of inflammation in healthy tissue, the acquisition of oncogenic KRAS mutations or sustained aberrant growth factor signaling can induce additional histological alterations. These alterations inhibit redifferentiation and promote further progression, ultimately resulting in precancerous pancreatic intraepithelial neoplasia (PanIN), which is the most common precursors of pancreatic cancer [29]. However, some studies have proposed alternative viewpoints. Alternative models propose that malignancies may arise directly from the ductal epithelium undergoing neoplasia without necessitating cell plasticity. In this process, ductal epithelium did not undergo cell plasticity [30]. Moreover, in human pancreas, shortened telomeres observed in PanINs were not found in ADM [31]. This supports the hypothesis that PanIN‐associated ADM arises secondary to PanIN lesions [31]. Therefore, resolving the precise role of ADM in pancreatic tumorigenesis will require the development of improved lineage‐tracing techniques and large‐scale clinical investigations.

#### 2.1.2 Metaplasia of the Esophagus and Stomach

In the stomach, the loss of acid‐secreting parietal cells and enzyme‐producing chief cells due to various acute and chronic gastric mucosal injuries—such as diphtheria infection, gastric peptic ulcers/erosions, and autoimmune or *H. pylori*–induced gastritis—can result in a metaplastic lesion [32]. Gastric cancer is frequently preceded by two principal forms of metaplasia: spasmolytic polypeptide‐expressing metaplasia (SPEM), involving the replacement of parietal and chief cells, and intestinal metaplasia, characterized by the ectopic emergence of MUC2‐expressing goblet cells [32, 33]. The current model of evolution from metaplasia to cancer suggests that after parietal cell loss, chief cells rapidly activate a cellular regeneration program called “paligenosis.” Paligenosis of chief cells begin when the differentiated chief cell reduces mTORC1 activity and increases autophagy to dismantle its mature cellular components. This process generates less differentiated, more plastic cells that subsequently activates metaplastic genes, specifically the SPEM markers TFF2 and MUC6, while reactivating mTORC1 to re‐enter the cell cycle [34, 35, 36]. Pyloric metaplasia is hypothesized to function as a temporary adaptive mechanism, providing a pool of regenerative cells capable of redifferentiating into mature cells or being replaced by normal gastric lineages once mucosal injury resolves. However, under conditions of sustained chronic injury or inflammation, this metaplastic phenotype can persist and expand, thereby facilitating the progression to intestinal metaplasia or dysplasia [32, 37]. Regarding the cellular lineage of these lesions, although current evidence indicates that chief cell reprogramming drives the majority of SPEM in acute settings, the potential contribution of mucous neck cells or isthmal progenitors cannot be fully excluded [38, 39]. In conclusion, although the precise mechanisms remain to be fully understood, gastric metaplasia arises from pre‐existing normal cells undergoing a plasticity program. Cell plasticity is a critical process that drives the transition from chronic inflammation to precancerous lesions and ultimately to malignancy in the stomach.

Barrett's esophagus is defined by the replacement of the native squamous mucosa in the distal esophagus with metaplastic columnar mucosa, a histological alteration that increases the risk of malignancy [28]. This specific tissue is termed specialized intestinal metaplasia (SIM), representing an incomplete form of intestinal metaplasia that contains a mixture of gastric‐type and intestinal‐type cells, notably goblet cells [28]. Nevertheless, regarding the mechanism of origin, there is no direct evidence to support the hypothesis that BE arises through the transdifferentiation of mature squamous cells [28, 40]. Instead, the development and maintenance of SIM are more likely driven by transcommitment, a process where immature progenitor cells, which retain the capacity to generate multiple lineages, undergo abnormal differentiation following chronic injury [28, 40]. However, due to the absence of a robust animal model, the specific cell of origin for Barrett metaplasia remains unknown [28, 40]. Although leading to esophageal adenocarcinoma, Barrett's esophagus might not perfectly align with the theme of plasticity as we are discussing. We look forward to in‐depth studies of this metaplastic process.

#### 2.1.3 Cell Plasticity in Melanoma Tumorigenesis

Beyond histologically observable metaplasia, research into the origins of cancer cells suggests that cell plasticity may also facilitate tumorigenesis through alternative mechanisms. In melanoma, the identity of the cell of origin remains a subject of ongoing debate [41]. Melanocytes originate from the neurogenic lineage, beginning as melanoblasts in the neural crest alongside other neurogenic precursors [41]. During embryogenesis, these cells disseminate to the distal anatomical regions and ultimately colonize these regions [41]. Approximately two‐thirds of melanomas arise on clinically healthy skin rather than from pre‐existing lesions. This observation implies that both nevi and malignant melanomas likely derive from the same mature epidermal melanocyte lineage [41, 42, 43]. Charles and colleagues used their zebrafish melanoma model and in vivo live imaging reporter to demonstrate that melanoma precursor cells reactivate an embryonic neural crest signature while simultaneously initiating a specific melanoma gene program [44]. Elevated ATAD2 expression has been shown to reactivate a progenitor‐like signature in melanocytes and enhance the ability of BRAF to initiate tumor formation [45]. Overall, these studies propose a model for the progression from melanocyte to melanoma. In this model, melanocytes lose their differentiated identity and acquire a neural and melanoblast‐like state characterized by stemness, which facilitates tumor development in the presence of oncogenic mutations.

#### 2.1.4 Cell Plasticity in Liver Cancer Tumorigenesis

Primary cancers arising in the liver mainly comprises hepatocellular carcinoma (HCC) and intrahepatic cholangiocarcinoma (ICC), which differ markedly in morphology, metastatic potential, and responses to therapy [46, 47]. It is logical to infer that these specific malignancies derive from their respective normal cellular lineages. However, previous studies have demonstrated that at least a subpopulation of ICCs derives directly from hepatocytes via mechanisms of cellular plasticity. Fan and colleagues utilized a hepatocyte fate‐tracing mouse model to demonstrate that overexpression of NICD and AKT can convert hepatocytes into atypical biliary cells, ultimately leading to lethal ICCs [48]. Tschaharganeh and colleagues demonstrated that loss of p53 function in mature hepatocytes induces a plastic cell state. Under the influence of lineage‐specific mutations in Notch signaling, these cells ultimately differentiate into ICCs [49]. Additionally, in a gene‐edited mouse HCC model, the necroptosis‐associated hepatic cytokine microenvironment can convert HCC to ICC, independent of the oncogenic drivers [46]. This transformation is regulated by the overexpression of Tbx3 and the loss of function of Prdm5 [46]. Indeed, recent studies have also identified other key regulators, including Yap‐Sox9 signaling, MYC, and the TF ETS1, which regulate hepatocyte plasticity and ICC tumorigenesis [50, 51]. Collectively, these findings highlight the significance of cell plasticity in liver cancer evolution and reveal the complex regulatory network of lineage commitment in liver cancer, as discussed earlier.

### 2.2 Cancer Cell Plasticity and Treatment Resistance

Indeed, despite the growing array of treatment options, therapeutic resistance remains a significant challenge, leading to treatment failure and disease recurrence. Under continuous selective pressure, tumor cell plasticity enables reversible shifts in cell identities that circumvent drug‐targeted pathways, driving tumor evolution [52]. Specifically, during drug exposure, cancer cells can utilize plasticity processes, including transdifferentiating into a resistant cell type or dedifferentiating into a stem‐like state to ultimately evade suppressive anticancer therapy [53].

#### 2.2.1 Neuroendocrine Transdifferentiation

Prostate cancer cell growth heavily dependent on heightened androgen receptor (AR) signaling. Therefore, targeted therapies aimed at inhibiting androgen synthesis or AR activation are widely employed. However, the transdifferentiation from adenocarcinoma to high‐grade neuroendocrine (NE) leads to castration‐resistant prostate cancer. Indeed, this is one of the most extensively studied models of lineage plasticity driving therapy resistance to date. Ku et al. and Mu et al. published back‐to‐back studies, demonstrating that the combined loss of TP53 and RB1 function, along with SOX2 overexpression, can induce castration‐resistant NE transdifferentiation in prostate adenocarcinoma [17, 54]. In addition, alterations in the expression and activity of pioneer TFs FOXAs and ASCLs, as well as epigenetic regulators EZH2, KMT2C and TET2, facilitate lineage plasticity [55, 56, 57, 58, 59]. Enhancing JAK/STAT inflammatory signaling and FGFR activity has also been demonstrated to enable NE transformation [60].

Furthermore, lung adenocarcinoma can also undergo NE transdifferentiation, resulting in NSCLC‐to‐SCLC lineage plasticity as a mechanism to evade targeted therapy. The identification of EGFR mutations and ALK rearrangements as actionable oncogenic drivers in NSCLC has revolutionized the treatment approach for patients with advanced‐stage disease, leading to a biomarker‐driven therapeutic paradigm [61]. Unlike GEMM‐based research, patient‐derived data on LUAD reveal NE transdifferentiation as a mechanism of resistance to targeted therapy. Pretreatment and post‐treatment resistant tumor samples from EGFR‐mutant or ALK‐positive patients demonstrate that SCLC transformation is one of the resistance mechanisms leading to tumor histological changes and independence from the original signaling axis [62, 63, 64]. Moreover, EGFR‐mutant NSCLC patients with concurrent RB1 and TP53 alterations have an increased risk of NE transdifferentiation, with 25% presenting with de novo SCLC or eventual small‐cell transformation [65]. Previous studies have also revealed that dysfunctions of epigenetic regulators and the PI3K/AKT and NOTCH pathways can promote lineage plasticity and therapy resistance [66, 67].

Moreover, the regulators governing NE transdifferentiation exhibit similarities in both lung cancer and prostate cancer. Park and colleagues demonstrated that the application of a defined set of oncogenic drivers is sufficient to reprogram normal human prostate and lung epithelial cells into prostate and lung SCNCs, respectively [68]. These data imply that NE cancers deriving from diverse epithelial lineages may be governed by conserved regulatory networks. In GEMM models, ASCL1 and ASCL2/POU2F3, well‐known NE TFs in SCLC, were demonstrated to drive prostate adenocarcinoma‐to‐ NE lineage plasticity [57]. Notably, Balanis et al. demonstrated that the convergence toward an SCN state is a pervasive phenomenon across diverse epithelial cancers, invariably correlating with adverse clinical outcomes [69]. Within this context, the convergence mechanism regulating pan‐cancer NE transdifferentiation highlights the potential for shared susceptibilities that can be targeted to overcome plasticity‐induced therapy resistance.

#### 2.2.2 Squamous Transdifferentiation

Squamous transdifferentiation represents an alternative mechanism of lineage plasticity, frequently emerging as the endpoint within another primary histological type [70]. Muscle‐invasive bladder cancers (MIBCs) can be categorized into luminal and basal (also known as squamous) subtypes. The squamous subtype of MIBC is demonstrated to exhibit resistance to chemotherapy and is associated with worse prognosis based on patient data [71]. Wang and colleagues developed an advanced primary and orthotopic MIBC mouse model, revealing that partial‐squamous differentiation is intrinsically linked to acquired chemoresistance in MIBC [72]. Their work demonstrated that partial‐squamous differentiation is intrinsically linked to acquired chemoresistance.

Additionally, estrogen receptor‐positive (ER+) breast cancer constitutes the predominant molecular subtype and generally exhibits sensitivity to endocrine therapy. However, the development of endocrine resistance is frequently driven by amplified phenotypic plasticity, a process characterized by the lineage switch of ER‐dependent luminal cells toward an ER‐independent basal‐like phenotype [73]. Previous research has revealed that dysfunction of the chromatin‐modifying complexes CoREST and SWI/SNF, as well as high‐order assemblies of TFs GATA3 and AP‐1, can facilitate the transformation process [73, 74, 75]. Moreover, in the patient‐derived xenograft (PDX), Labrecque et al. identified a squamous differentiation cell state in the castration‐resistant prostate cancer [76].

In lung adenocarcinoma, adeno‐squamous transdifferentiation is another mechanism leading to target therapy resistance. Schoenfeld and colleagues reported that 5 of 62 patients with EGFR‐mutant lung adenocarcinoma, progressing on first‐line Osimertinib, exhibited squamous transdifferentiation [77]. In ALK‐rearranged lung adenocarcinoma patients treated with ALK tyrosine kinase inhibitors, squamous lineage plasticity also drives therapy resistance [78]. Early clinical results with recently developed KRAS inhibitors also revealed that histologic transformation to squamous‐cell carcinoma can confer resistance to these inhibitors [79]. Mouse model research demonstrated that the LKB mutant and ELF5–ΔNp63 axis are important regulators of KRAS inhibition‐resistant lineage plasticity [19]. Moreover, pancreatic ductal adenocarcinoma can acquire characteristics of squamous (also known as basal) epithelial cells during disease progression. In human samples and GEMMs, previous studies revealed a complex regulatory network involved in this transformation [10, 80, 81]. Basal‐like pancreatic cancer is demonstrated to be more aggressive and resistant to chemotherapy and target therapy, leading to a worse prognosis compared to the classical type [10, 80, 82]. However, Dilly et al. and Singhal et al. demonstrated the basal‐type pancreatic cancer is sensitive to the KRAS inhibitor therapy, and the classic‐state PDAC drive the target therapy resistance [83, 84]. This result is opposite to the finding that squamous state leads to KRAS inhibitor resistance chemotherapy resistance in PDAC. Consequently, the combination of chemotherapy with KRAS inhibition represents a viable strategy to concurrently target distinct resistance modalities

#### 2.2.3 Epithelial–Mesenchymal Transdifferentiation

Epithelial–mesenchymal transdifferentiation is a classical cellular plasticity program that is extensively studied in embryogenesis, wound healing, malignant progression, and tumor metastasis [26]. Indeed, the EMT program in tumors is rarely binary and often leads to a dynamic equilibrium between mesenchymal and epithelial phenotype. In this section, we review recent advances in understanding the key roles of EMT in tumor therapy resistance. In the next part, we will discuss the various processes of EMT‐induced metastasis.

Malignant cells undergoing EMT can acquire resistance to various chemotherapeutic drugs [85]. Previous studies revealed that EMT expression profile in the tumor is associated with worse prognosis [86, 87]. The EMT‐regulating TF Snail has been demonstrated to inhibit p53‐mediated apoptosis and disrupt the cell cycle, thereby contributing to resistance to cell death [88, 89]. EMT can also selectively represses apoptosis signaling through the death receptors DR4 and DR5 [90]. In various cancer cell lines, overexpression of EMT‐TFs induces EMT and contributes to resistance to chemotherapy [91, 92, 93]. Employing mouse models of pancreatic and lung cancer, Zheng et al. and Fischer et al. independently demonstrated that the acquisition of chemotherapeutic resistance in both primary and metastatic tumor cells is driven by an EMT‐dependent mechanism [85, 94]. Furthermore, recent evidence identified RHOJ—a small GTPase expressed in cells undergoing EMT—as a critical mediator that accelerates the repair of chemotherapy‐induced DNA lesions, thereby governing EMT‐associated resistance [95].

In addition, the role of EMT in mediating resistance to EGFR TKIs in EGFR‐mutant lung cancer patients has been extensively researched [96]. In patients with drug‐resistant NSCLCs carrying EGFR mutations, EMT has been identified through systematic genetic and histological analyses [97]. Several studies on EGFR‐mutated NSCLC cell lines also suggest the role of EMT in acquired resistance to EGFR‐TKIs [98, 99, 100, 101]. Mechanistically, activation of AXL signaling in the EMT state can lead to EGFR‐TKI resistance through bypass activation of EGFR downstream molecules [102, 103, 104]. Moreover, FOXM1 and ASCL1, which is not regard as classical EMT‐TFs, is demonstrated to induce EMT‐associated EGFR TKI resistance in lung cancer. Indeed, two recent studies revealed that targeting CD70 and Aurora B kinase, respectively, in EGFR‐mutant cell lines can overcome EGFR‐TKI resistance beyond EMT [105, 106].

Indeed, EMT can also drive acquired resistance to immunotherapy through tumor cell modulation and microenvironment reprogramming [107, 108]. On the one hand, through tumor‐intrinsic mechanisms, mesenchymal‐like state cancer cells have been shown to respond poorly to immunotherapy in patients treated with PD‐1 inhibitors [109]. EMT programs can enhance cancer cell autophagy, enabling tumor cells to evade T cell‐mediated lysis and acquire resistance to immunotherapy [110, 111]. Moreover, cancer cells in a mesenchymal‐like state can develop defective antigen presentation by expressing reduced levels of HLA, hindering their recognition by the immune system [112, 113]. Furthermore, EMT induces epigenetic and transcriptional silencing of interferon regulatory factor 6 (IRF6), suppressing the responsiveness of tumor cells to TNF‐α‐mediated T‐cell killing [114]. On the other hand, activation of EMT can contribute to the generation of an immunosuppressive microenvironment, further supporting tumor immune evasion [115]. Kudo‐Saito et al. revealed that melanoma cells undergoing Snail‐induced EMT can promote regulatory T cells and impair dendritic cells via TSP1 production [116]. Snail activation can also increase the expression of CCL2 and CCL5, leading to the recruitment of tumor‐associated macrophages (TAMs), which inhibit T cell migration and activation [117]. Moreover, factors derived from mesenchymal‐like cancer cells, such as CD73, CSF1, and SPP1, can further suppress CD8 lymphocyte activation [118].

### 2.3 Cancer Cell Plasticity and Metastasis

Metastasis, the spread of cancer cells to distant organs from their site of origin, represents the final and most lethal stage of tumor evolution, accounting for the majority of cancer‐related deaths [119]. Previous studies have outlined metastasis as a process comprising three stages—dissemination, dormancy, and colonization—during which cancer cells invade tissues, survive transit, and establish colonies in distant organs [24, 119, 120]. During the metastatic cascade, plasticity allows cancer cells to adapt to various stresses and respond dynamically to changes within the TME. In this section, we review the critical roles of cellular plasticity in facilitating the distinct phases of metastasis.

#### 2.3.1 Cell Plasticity and Dissemination of Tumor Cells

EMT is the most prominent example of cellular plasticity contributing to the initiation of metastasis [121]. The EMT process leads to the deficiency of apical–basal polarity and the loss of epithelial cell adhesion molecules, ultimately enhancing cancer cells' ability to migrate and invade surrounding tissues [122]. Yang and colleagues were the first to reveal that Twist is a crucial regulator of the EMT process and tumor metastasis [123]. Stemmler et al. suggest that EMT‐TFs, including Twist, establish a complex and dynamic regulatory network that promotes metastasis [124]. Moreover, hypoxia‐inducible factor‐1alpha (HIF‐1alpha) caused by intratumoral hypoxia can also promote EMT and metastasis [125]. Immune cells within the TME and mechanical cues from the extracellular matrix have also been identified as cell‐extrinsic mechanisms that facilitate EMT‐induced tumor metastasis [126, 127, 128, 129].

Recent studies indicate that EMT is not merely a binary transition between epithelial and mesenchymal phenotypes [122, 130]. Instead, cells can exhibit epithelial–mesenchymal plasticity (EMP), characterized by the simultaneous expression of both epithelial markers (such as cytokeratin) and mesenchymal features (such as enhanced motility) and transition between intermediate epithelial/mesenchymal (E/M) states along a continuous epithelial–mesenchymal spectrum [130]. Cancer cells undergoing EMP, while existing in a hybrid EMT (hEMT) state, are demonstrated to possess the highest metastasis potential [131, 132]. Indeed, through the use of single‐cell sequencing and innovative lineage‐tracing techniques in mouse models, cancer cells exhibiting EMP characteristics have been identified as the primary metastatic‐initiating cells [23, 133]. Moreover, Pastushenko and colleagues revealed that the deletion of Fat1 activates the CAMK2–CD44–SRC axis, while simultaneously inhibiting EZH2 and promoting SOX2 expression. This dual mechanism supports both mesenchymal and epithelial states concurrently, reinforcing EMP and tumor metastasis [134]. Epigenetic regulator MLL3 loss of function can also induce EMP‐associated metastasis through increasing IFNγ signaling [135].

#### 2.3.2 Cell Plasticity and Colonization of Tumor Cells

Indeed, some researchers debate the involvement of EMT in the metastasis process due to the lack of a mesenchymal phenotype in human carcinoma metastases [122, 136]. Chaffer and colleagues demonstrated higher epithelial characteristics were associated with increased bone and soft tissue colonization [137]. Chao et al. revealed that the epithelial marker E‐cadherin can be re‐expressed in secondary metastatic organs driven by microenvironment‐induced mesenchymal–epithelial transition (MET) [138]. These findings collectively reveal the essential role of epithelial characteristics in metastasis and MET following EMT is necessary for successful colonization and metastasis [139]. In the PDAC metastasis mouse model, P120CTN‐mediated stabilization of membranous E‐cadherin can promote tumor cell MET and liver metastasis [140]. Moreover, the loss of Twist or Prrx1, which are inducers of EMT, has been demonstrated to revert cancer cells to the epithelial phenotype and contribute to metastasis [141, 142]. Previous studies suggest that cancers exhibit a metastatic organ tropism, where different types of cancer often show a preference for colonization in specific organs [143]. Specific stromal components in distant organs can induce tumor cell plasticity and colonization. In the prostate cancer bone metastasis model, E‐selectin within bone vascular niche can bind to cancer cell directly, facilitating Wnt signaling activation and MET‐induced tumor bone metastasis [144]. Recent in vivo interaction screening studies have demonstrated that plexin B2 expressed by hepatocytes interacts with class IV semaphorins on tumor cells, leading to the upregulation of KLF4, which subsequently promote MET and facilitates tumor metastasis [145].

#### 2.3.3 Cell Plasticity and Dormancy of Tumor Cells

After dissemination and extravasation, certain disseminated tumor cells (DTCs) manage to colonize distant organs and activate proliferative programs, eventually leading to the formation of clinically detectable metastatic lesions [119]. However, in some cases, DTCs lack the full suite of required adaptations can survive in distant organs but fail to sustain active proliferation, entering into a dormant state [146]. These dormant cells can persist for extended periods, potentially reactivating later to drive tumor recurrence. Recent studies demonstrate that the EMP program is linked to the dormancy and recurrence of DTCs [24].

Specially, in metastatic breast cancer PDX models, the low metastatic burden dormancy cell state enriched a high mesenchymal and survival signature, meanwhile the DTCs from high metastatic burden animals exhibited an epithelial and proliferation signature [147]. Harper et al. demonstrated that early dissemination mammary cancer cells can intravasate and lodge in target organs through a Wnt‐dependent EMT‐like program [148]. Moreover, Nobre and colleagues revealed that the TF ZFP281 induces a mesenchymal program in early disseminated breast cancer cells, locking them in a dormant state and preventing the transition to an epithelial‐like proliferative state and metastatic outgrowth [149]. Indeed, Aouad and colleagues used their PDX model of ER+ breast cancer to demonstrate the critical role of EMP in metastasis dormancy and recurrence. They found that mesenchymal‐like dormant disseminated ER+ breast cancer cells proliferated at a slower rate. However, when these cells re‐expressed E‐cadherin, a key epithelial marker, they transitioned to a more epithelial state, which led to metastatic outgrowth [150]. In addition to EMP, in ER+ breast cancer, the kinase MSK1 plays a crucial role in regulating the luminal cell program that defines the metastatic dormancy state. Loss of MSK1 function impairs breast cancer cell differentiation, enhancing homing and growth capabilities and leading to early metastasis [151].

### 2.4 Cell Plasticity and Cancer Stem Cells

In the above paragraph, we primarily discuss the transdifferentiation aspect of cellular plasticity in the complex and dynamic process of cancer evolution. Moreover, among unlocking phenotypic plasticity, in addition to transdifferentiation, the aspects of dedifferentiation and blocked differentiation, which can induce progenitor or stem cancer cell states, are also extensively researched in cancer evolution [8]. Indeed, the progenitor‐like, highly plastic cell state, commonly referred to as CSCs, has been a subject of research since the 19th century. A recent comprehensive review has meticulously summarized the defining characteristics and emerging hallmarks of CSCs, summarizing their role in cancer progression and therapeutic resistance [152]. In this section, we will briefly discuss the relationship between CSCs and cellular plasticity, highlighting their critical role in tumor evolution.

#### 2.4.1 Cell Stemness: From Population to Plastic Cell State

The classical model of CSCs described these cells as a rare, relatively quiescent subpopulation exhibiting constrained plasticity. Residing at the hierarchical apex, CSCs are characterized by their intrinsic capacity for self‐renewal and their potential to differentiate into the heterogeneous cellular architecture of the tumor [153]. This conceptual framework is predicated, in part, upon the principles governing normal stem cell biology. Within a rigid hierarchical architecture, stem cells give rise to progeny with progressively restricted proliferative and differentiative potential, adhering to an irreversible trajectory of lineage commitment [6]. Consequently, the differentiated progeny would lack the capacity to revert to a stem cell state, maintaining a clear distinction between stem cells and differentiated cells. However, in the context of wound healing and tissue regeneration, differentiated somatic cells may acquire plasticity, thereby replenishing the stem cell pool to facilitate tissue repair [154]. In various cancer types, researchers have observed differentiated cancer cells reverting to the CSC state to replenish the CSC pool and induce tumor regrowth [155, 156, 157]. Recent studies suggest that CSCs are not a distinct, static cell type with inherent self‐renewal and differentiation capabilities, but rather represent dynamic and fluid cell states. Differentiated cancer cells can transition into these CSC‐like states in response to microenvironmental changes or therapeutic pressures, contributing to malignant features and tumor evolution [152, 158, 159]. Mechanistically, CSC cell state plasticity can be induced by cell‐intrinsic and cell‐extrinsic signaling resembling the transdifferentiation process mentioned above.

Cell‐intrinsic signaling is essential in mature cell differentiation and CSC plasticity. Zhu and colleagues demonstrated that tetraspanin‐8, interacting with the SHH‐PTCH1 complex, can activate Sonic Hedgehog signaling, promoting breast cancer stemness [160]. In mouse models and patient samples of breast cancer, overexpression of the TF SOX10 can induce a CSC state by activating genes that regulate neural crest cell identity [161]. Moreover, the nuclear receptor RXRG is a key driver of the neural crest stem cell (NCSC)‐like state in melanoma [162]. In colorectal cancer cell lines, TRIB3 interacts with β‐catenin and TCF4, leading to the upregulation of genes linked to CSC traits, thereby promoting the presence of LGR5‐positive colorectal CSCs [163]. In PDAC, Bcl3 has been shown to suppress the CSC population and promote cellular differentiation. Loss of BCL3 leads to an expansion of the CSC compartment, which is associated with poorer survival outcomes [164]. Deng et al. revealed that ectopic JAK‐STAT activation in prostate cancer can drive resistance to AR‐targeted therapies by promoting stem‐like subclones with multilineage transcriptional programs, rather than restricting transition to NE‐like lineage [165].

CSCs reside in the niche, which refers to the microenvironment that sustains self‐renewal and restricts premature differentiation of the stem cell pool [166]. The heterogenous CSC niche is essential for CSC plasticity [167]. CAFs within the tumor stroma play a crucial role in supporting CSC niche. In mouse models and PDX of breast cancer, CAFs produce fibrillar collagen and FGF5, creating a supportive niche that facilitates the acquisition of the CSC phenotype [168]. Su and colleagues identified and isolated a specific subset of CAFs characterized by the surface markers CD10 and GPR77 [169]. This subset provides a survival niche for CSCs, contributing to poor outcomes in multiple cohorts of breast and lung cancer patients. In addition, Ma et al. discovered a specific CAF subpopulation in bladder cancer, induced by interferon signaling, that promotes cancer stemness by upregulating the urea transporter SLC14A1 and activating the WNT5A paracrine signaling pathway [170]. Furthermore, immune cells in the microenvironment are also key components of the CSC niche. TAMs can upregulate the expression of EphA4, which binds to receptors on tumor cells, activating Src and NF‐κB. NF‐κB then induces the secretion of various cytokines, promoting the CSC state [171]. Beziaud and colleagues revealed that IFNγ secreted by activated T cells can directly drive the transition of breast cancer cells to a CSC state [172]. Recent studies have also highlighted the involvement of various cell types in the CSC niche. In breast cancer mouse model, nuclear prelamin A recognition factor (NARF) induced by the hypoxia microenvironment facilitates the expression or activity of stem cell pluripotency factors including NANOG, KLF4, and SOX2, leading to CSC activation [173]. Pietras et al. demonstrated that in the glioma perivascular niche, Osteopontin‐CD44 signaling regulates CSC phenotype transitions by enhancing HIF‐2α activity under normoxic conditions [174].

#### 2.4.2 Plastic Model of Intratumor Transcriptional Heterogeneity

Indeed, the critical role of CSCs in tumor initiation, progression, metastasis, and therapy resistance has been comprehensively summarized in a recent insightful review [152]. When cancer stemness is viewed as a cell state rather than a distinct cell type, CSC plasticity can be integrated into the theoretical framework of cell plasticity, as discussed earlier. CSC plasticity can be induced by enabling characteristics and facilitates various cancer hallmarks, serving as a critical intermediate process for understanding dynamic tumor evolution. However, between CSCs and cell plasticity, a key controversy persists: which program plays the dominant role in driving intratumor transcriptional heterogeneity [158, 175]?

**FIGURE 2:**
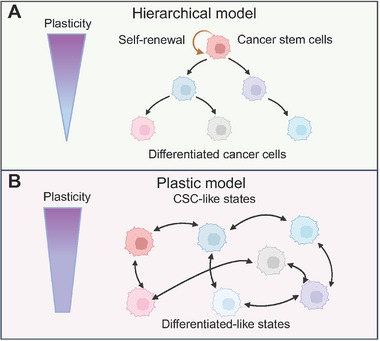
Two models of intratumor heterogeneity. In the hierarchical model, CSCs are positioned at the apex of the cellular network, possessing the ability to self‐renew and generate hierarchical differentiated cancer cell populations. With progressive differentiation, cancer cells gradually lose plasticity and are unable to revert to a less differentiated state. In the plastic model, cellular hierarchy is not strictly fixed but is instead defined by the dynamic ability of cells to transition and interconvert between various states. The different cancer cell populations have various degrees of plasticity. Abbreviations: CSC, cancer stem cell.

The tumor is a highly complex organ, made up of a diverse array of cell types and cellular states, which contribute to intratumor heterogeneity [8]. Certainly, one aspect of this phenotypic heterogeneity arises from chronic or episodic genomic instability, leading to genetic diversity among tumor cells. Moreover, growing evidence highlights the importance of epigenetic diversity, which occurs independently of genetic mutations. As a defining hallmark, the multipotency of CSCs enables their differentiation and contributes to intratumor heterogeneity, representing the classic view of tumor progression (Figure 2A) [152]. Under the hierarchical CSC model, the CSC population proliferates and “rolls down” to generate and sustain diverse lineages of differentiated cancer cells, thereby driving intratumor heterogeneity [176]. The hierarchical framework is supported by investigations across diverse malignancies. Specifically, through the implementation of a DNA‐barcoded primary glioblastoma cell xenotransplantation model, Chen et al. demonstrated that slow‐cycling stem‐like cells function as the origin of a rapidly proliferating progenitor population exhibiting extensive self‐renewal capacity, which subsequently differentiates into diverse nonproliferative cells [177]. Tirosh and colleagues utilized scRNA‐seq to analyze cancer cells from human oligodendroglioma samples, revealing that CSCs drive the formation of a heterogeneous cell population through a developmental hierarchy that mirrors normal differentiation along glial lineages [178]. Moreover, Wang et al. identified a tetraspanin CD9‐high CSC population that gives rise to PDAC heterogeneity and initiates tumor development [179].

However, as we mentioned above, recent studies emphasize that cancer cell stemness is a dynamic cell state. A unidirectional, hierarchical CSC model seems insufficient to account for the dynamic plasticity within heterogeneous cancer cell populations. Indeed, in colon cancer, after LGR5+ CSC ablation, Lgr5− cancer cells dedifferentiate to the CSC state and induce the tumor recurrence [155, 180]. Karras and colleagues demonstrated a nonrandom spatial distribution of CSCs in mouse melanoma, suggesting that dedifferentiation, driven by signals from the endothelium, can produce CSCs that occupy the apex in the cellular hierarchy [133]. Moreover, Neftel et al. identified four primary cellular states in glioblastoma, each resembling different neural cell types, using scRNA‐seq data from fresh human tumor samples [181]. In the lineage‐tracing experiment with the PDX model, plasticity was demonstrated between these four states, with multiple possible transitions [181]. These results suggest that cancer cell plasticity, including CSC plasticity, leads to the intratumor heterogeneity (Figure 2B). Additionally, recent studies utilizing spatial omics have revealed that intratumor heterogeneity can result from heterogeneity in the signals that cells receive [182, 183]. These studies suggest that the spatial architecture of tumors—encompassing intercellular interactions and the diffusion gradients of oxygen and nutrients—modulates the plastic functional roles of cancer cell states, thereby contributing to intratumor transcriptional heterogeneity [182, 183].

Indeed, Patel et al. recently posited a developmental constraint model [184]. They suggest that, similar to the CSC model, the various cell states within a given cancer type are interconnected along a hierarchy [184]. They constrain the development of cancer cell subpopulations to states that are directly connected within the original cells' normal developmental hierarchy, suggesting that transitions occur only between closely related developmental stages [184]. In this model, CSCs are not considered a distinct, privileged population responsible for intratumor heterogeneity. Instead, the primary driver of heterogeneity is the developmental‐constrained plasticity of cell states, which plays a central role in the evolution of diverse subpopulations [184]. Indeed, this model acknowledges the contribution of both forward progression toward differentiated states and the reverse process of dedifferentiation into stem‐like states in tumor progression. In conclusion, their research provides a strategy for integrating CSC state into cellular plasticity to elucidate heterogenous composition of the malignant cell states.

## 3 Enabling Characteristics of Cell Plasticity in Cancer

**FIGURE 3:**
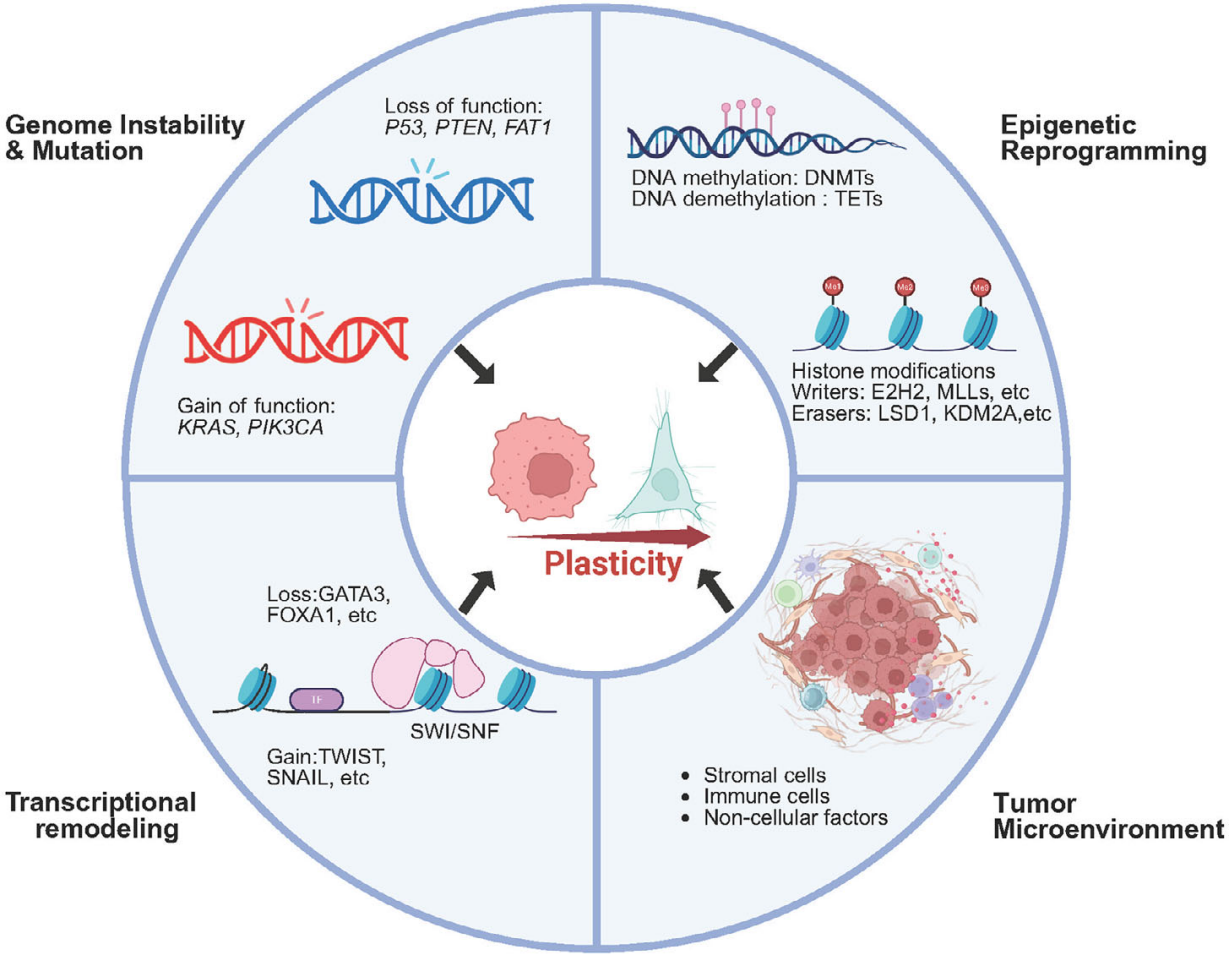
Enabling characteristics contribute to cancer cell plasticity. Cancer cell plasticity can arise as a downstream consequence of intrinsic factors, such as genome instability, epigenetic reprogramming, and transcriptional remodeling, or from extrinsic influences within the tumor microenvironment.

Having established the essential role of cellular plasticity in tumor progression, metastasis, and therapeutic resistance, we now examine the complex mechanisms governing this process. Cellular plasticity is not driven by a single pathway but arises from the interplay of key characteristics fundamental to the hallmarks of cancer. Accordingly, we will first examine cell‐intrinsic drivers—including genomic instability, epigenetic reprogramming, and transcriptional remodeling—before exploring how extrinsic signals from the tumor microenvironment induce and sustain this plastic potential (Figure 3).

### 3.1 Genome Instability and Mutation

As an important enabling characteristic, genome instability leads to driver (and passenger) mutations that confer functional capabilities fundamental to cancer. The genetic fluctuation of key signaling pathways is a significant cause of cell plasticity during tumor evolution [185].

TP53, a frequently mutated cancer suppressor gene in human cancers, ensures lineage commitment and inhibits the plasticity of cancer cells, which are crucial aspects of its tumor‐suppressive functions [186, 187]. In alveolar cells, p53 loss of function results in persistence of inappropriate cell line divergence from lung lineage identity associated with adenocarcinoma [188]. Loss of p53 can also promote the plasticity and give rise to more diversity and more aggressive cell states in lung cancer evolution [189]. In luminal progenitors which are origin of triple negative breast cancer (TNBC), previous researches using mouse model and human tissues reveals that TP53 loss of function, along with BRCA1 mutation, induces the basal/mesenchymal transition and may result in basal‐like tumor development [190, 191]. In hepatocytes, p53 loss facilitates cell plasticity and dedifferentiation into nestin‐positive progenitor‐like cells, which can transform into HCC or cholangiocarcinoma under additional lineage‐specific mutations [49, 192]. TP53 loss of function is also demonstrated as an essential factor in prostate cancer NE lineage plasticity [17, 54].

In addition, Fat1 deletion induces a plastic epithelial–mesenchymal transition (EMT) state in squamous cell carcinoma [134]. Oncogenic KRAS mutation could revert differentiated and quiescent alveolar epithelial type II (AT2) cells back to its original stem cell state, leading to adenomatous transformation [193]. The KRAS mutation induces premalignant ADM in development of pancreatic ductal adenocarcinoma [29, 194]. PIK3CA, an oncogene involved in the PI3K/AKT signaling pathway, promotes the dedifferentiation of mammary cells into a plastic stem‐like state during the early stages of tumor initiation through its gain‐of‐function mutations [195, 196]. Moreover, PTEN, a tumor suppressor gene within the same pathway, contributes to increased cell plasticity and histopathologic heterogeneity in NE tumors due to its loss‐of‐function mutations [197]. Recent study indicates that, in addition to mutations, extrachromosomal DNA (ecDNA) can drive gain‐of‐function in key oncogenes and promote plasticity in PDAC [198].

On the other hand, gene mutation can also prevent lineage plasticity. Analysis of biopsies from heavily pretreated, ER‐positive breast cancers revealed tumors lacking detectable *ESR1* mutations frequently adopted a mixed‐lineage phenotype, indicative of lineage plasticity, whereas *ESR1*‐mutant tumors largely preserved their luminal identity and ERα dependency [199]. Furthermore, distinct classes of mutations can drive different cell state. Eyunni et al. demonstrate that n prostate cancer, the multifunctional oncogene FOXA1 has two distinct roles that are driven by different classes of mutations: one set promotes primary AR‐dependent tumorigenesis, while another confers intraluminal plasticity and induces AR‐resistant progression [200].

### 3.2 Epigenetic Reprogramming

As an enabling characteristic, epigenetic reprogramming has been identified as a key mechanism driving cellular plasticity [14]. Broadly, Epigenetic reprogramming encompasses any heritable molecular modifications that cause phenotypic alterations without altering the DNA sequence [185]. In this section, we primarily discuss DNA methylation and post‐translational modifications of histones.

Post‐translational modifications of histones refer to the addition and removal of histone codes. Enzymes responsible for controlling the processes are key regulators of lineage plasticity. Histone modification writers can add specific chemical groups to histones, establishing epigenetic marks that regulate chromatin structure and gene expression. Polycomb repressive complexes 2 (PRC2) promotes the deposition of histone H3 lysine 27 trimethylation (H3K27me3) [201]. PRC2 loss of function leads to a quasimesenchymal state that is associated with highly metastatic capabilities and poor survival of patients with breast cancer [201]. Activation of E2H2, the enzymatic subunit of PRC2, drives adenocarcinoma to NE lineage plasticity in prostate cancer and “classical” and “basal‐like” transmission in pancreatic ductal adenocarcinoma [80, 202]. Moreover, two recent studies respectively demonstrated that the loss of function of the histone methyltransferases MLL2 or MLL3 promotes EMT in breast cancer [135, 201]. MLL3 can also drive double‐negative transdifferentiation and AR‐resistance in prostate cancer. Another histone methyltransferase NSD3 promotes EMT and CSC like state through methylation of H3K36 in the breast cancer progression [203]. In addition to histone modification writers, Nguyen et al. reported that lactate can increase histone acetylation and active MYC, ultimately driving lineage plasticity in colon cancer [204].

Furthermore, the histone modification erasers majoring in removing the marks left by writers can also regulate the cancer cell plasticity. Lysine‐specific demethylase 1 is demonstrated to promote prostate cancer NE lineage plasticity through its H3K9 demethylase function [205, 206]. LSD1‐mediated demethylation of H3K4me2 on the E‐cadherin (CDH1) promoter can also active EMT in breast cancer [207]. KDM6A demethylase the H3K27, it suppresses pancreatic cancer through maintaining differentiated acinar cell programs and its loss of function induces adeno to squamous plasticity in pancreatic cancer [81, 208]. Previous study also reveals KDM6A as an epigenetic regulator that controls ASCL1 to NEUROD1 subtype plasticity of small‐cell lung cancer [209]. Indeed, Yuan et al. revealed that the regulation of histone mark H3K36me2 can drive the EMP of PDAC through reprogramming of enhancers associated with key EMT genes [210]. Specifically, loss of the histone methyltransferase NSD2 inhibited EMT, while loss of the demethylase KDM2A suppressed MET [210]. In conclusion, this study underscores the global regulation of post‐translational histone modifications as a critical epigenome mechanism underlying cancer cell plasticity.

Another key epigenetic mechanism influencing the cancer cell plasticity is DNA methylation. This modification is introduced by DNA methyltransferases (DNMTs) and removed by ten–eleven translocation (TET) proteins. DNMT1, in particular, has emerged as a critical regulator of cellular plasticity. Several studies have demonstrated that DNMT1‐induced methylation of key regulatory factors facilitates the transition of breast CSCs [211, 212, 213]. Moreover, DNMT1 can also promote EMT in breast cancer through methylating the wwc1 promoter [214]. Nevertheless, in liver cancer, DNMT1 loss of function mediated demethylation of the BEX1 promoter leads to activation of downstream signaling and maintain the high plastic stem‐like subtype [215]. In addition, mutant DNMT3A potentiates transactivation of stemness genes and drives stemness of leukemia cells [216].

As for TET proteins, in cervical cancer, TET1 activation suppresses EMT [217, 218]. TET1 can bind to and catalyze the demethylation of promoters, thereby activating the transcription of SFRP2, ultimately inhibiting EMT in pancreatic tumors [219]. Additionally, TET2 loss of function facilitates the squamous lineage plasticity of PDAC [220]. Whereas TET2 activation is demonstrated an epigenetic mechanism regulating tumor NE lineage plasticity in prostate cancer [58].

In conclusion, post‐translational modifications of histones and DNA methylation constitute a complex regulatory network that controls cell fate in cancer. Disruptions in these processes can lead to cellular plasticity without altering the DNA sequence. Furthermore, alterations in lineage TFs and chromatin remodeling can also drive cell plasticity through nongenetic mechanisms.

### 3.3 Transcriptional Remodeling

Chromatin‐remodeling complexes play a crucial role in repositioning, ejecting, or restructuring tightly bound nucleosomes [221, 222]. They operate in conjunction with TFs to modulate chromatin structure, thereby influencing transcription and regulating gene expression in normal physiological and cancerous contexts [221, 222].

The SWI/SNF family of chromatin‐remodeling complexes, also referred to as BRG1/BRM‐associated factor (BAF) complexes, are critical regulators of cancer cell plasticity. AT‐rich interaction domain‐containing protein 1A (ARID1A) is a component of SWI/SNF. It suppresses precancer ADM and cancer EMT in pancreatic cancer [223, 224]. ARID1A loss of function promotes luminal to basal‐like cell lineage plasticity in breast cancer [75] and EMT phenotype in pancreatic cancer [225]. Another SWI/SNF core subunit SMARCA4 also participates the cancer cell plasticity. Previous researches reveal that SMARCA4 is the key factor of NE lineage plasticity in prostate cancer and SCLC [226, 227]. Nevertheless, there is also a study suggesting that the loss of SMARCA4 may drive plastic cell state in lung adenocarcinoma [228]. Additionally, Patrizia and colleagues demonstrate that inactivation of the chromatin‐remodeling enzyme ATRX disrupts colonic epithelial identity and promotes the emergence of highly plastic mesenchymal and squamous‐like cell states [229].

During embryonic development, lineage TFs play crucial roles in directing organ specification and establishing tissue patterns [230]. Conversely, in cancer, this well‐regulated process becomes disrupted, altering transcriptional pathways to induce cell plasticity [14, 231]. The abnormal activation of lineage‐specific TFs can lead to plasticity in tumor evolution. Core EMT factors such as ZEB1, ZEB2, SNAI1, SNAI2, and TWIST1 are notable examples. Their aberrant gain‐of‐function enables epithelial cancer cells to acquire mesenchymal‐like characteristics [11]. As key pluripotency TFs in embryonic stem cells, the activation of SOX2 and OCT4 can induce NE lineage plasticity in prostate cancer [17, 232]. Additionally, KLF4 is crucial for acinar‐to‐ductal plasticity during early pancreatic carcinogenesis [233].

Moreover, loss or reprogramming of specific TFs that define the original tumor lineage can also facilitate cancer cell lineage plasticity. In human non–small‐cell lung cancers, loss of lung lineage‐specifying TF NKX2‐1 leads to plastic tumors bearing features of various gut tissues [234]. NKX2‐1 can also drive NE transdifferentiation of prostate cancer via 3D chromatin remodeling [235]. In addition, Lkb1‐deficient induces the lineage plasticity of lung adenocarcinoma transdifferentiating to lung squamous cell carcinoma. In pancreatic NE tumors, loss of Meis2 TF enables benign islet tumors to invasive, metastasis‐like primary (MLP) tumors lineage plasticity [236]. Loss of TFAP2 neural crest TF in melanomas is associated with a plastic cell state, leading to transmission of proliferative to invasive subpopulations [237]. Combining loss of GATA3 and elevation of AP1 in breast cancer can result in luminal to basal phenotypic plasticity [73]. Furthermore, previous researches reveal that redistribution of key prostate cancer–associated TFs, AR and FOXA1, from epithelial‐associated to NE‐associated regulatory elements promote NE cell‐like lineage plasticity [73, 238, 239, 240]. Moreover, cell‐type‐specific transcriptional repressors actively maintain cell identity, and the loss of function of these repressors can also promote unwanted cell plasticity [241, 242].

As mentioned above, intrinsic enabling characteristics of cancer cells contribute to their plasticity. Additionally, cancers are complex ecosystems composed of tumor cells, various noncancerous cells, and an altered extracellular matrix [243]. Interactions between tumor cells and the TME are considered a form of cancer cell‐extrinsic enabling characteristic [185]. In the following section, we will discuss how the cross talk between TME and cancer cell enables plasticity.

### 3.4 Tumor Microenvironment

The microenvironment in which a tumor grows can have a profound impact on cancer cell lineage plasticity. Interactions between tumor cells and stromal cells, immune cells, and noncellular factors are crucial extrinsic mechanisms that promote cell state transitions and the emergence of highly plastic cell populations.

Cancer‐associated fibroblasts (CAFs) constitute a major component of the tumor stroma and are primarily involved in tumor maintenance and promotion through a variety of mechanisms [244]. Previous studies reveal that CAF regulate cancer cell plasticity through cytokine secretion [244]. Nallasamy and colleagues demonstrate that long‐term exposure of pancreatic cancer cells to conditioned media derived from CAFs promote the acquisition of stem‐like properties [245]. Another study revealed CAF‐secretion VEGFA and ITGB1 are the key factors facilitating pancreatic cancer cell EMT [246]. In precancer ADM of pancreatic acinar cells, CAF active laminin α5/integrinα4/stat3 axis and induce cell plasticity [247]. In addition, in NSCLC and ICC, CAF promotes the high plastic CSC through IGF‐II/IGF1R signaling [248] and FGF5 expression together with production of fibrillar collagen [168], respectively. Cartilage oligomeric matrix protein from CAF is also demonstrated to induce EMT of HCC [249]. Moreover, CAF can directly transfer exosomes to CRC cells, leading to Wnt/β‐catenin pathway activation and mitochondrial apoptosis inhibition, finally induce EMT and stemness of CRC [250]. Besides acting along, CAF can synergize with other cells in the microenvironment. In ICC, CAF recruits and educates myeloid‐derived suppressor cells that activating 5‐LO/LTB4–BLT2 axis, finally facilitates cancer stemness [251]. Indeed, inflammation is another important microenvironmental factor that is intimately connected to lineage plasticity.

The mechanisms through which TAMs contribute to tumor plasticity have been extensively investigated [252]. In glioblastoma, several studies have demonstrated that TAMs could promote cancer cell mesenchymal‐like transmission within microenvironment [109, 253, 254]. This effect is mediated through mechanisms such as macrophage‐derived oncostatin M (OSM) [253] and TNF‐α/NF‐κB‐activation [254]. In NSCLC, tissue‐resident macrophages accumulate near tumor cells early during tumor formation, where they promote EMT [255]. TAMs can also induce EMT of kidney cancer through expressing IL1B at the tumor‐normal interface [256]. In addition, TAMs enhance the tumor cell stemness in glioblastoma via integrin αvβ5‐Src‐Stat3 signaling [257] and in breast cancer through Notch‐Jagged signaling [258]. Moreover, Chan and colleagues demonstrated that a profibrotic monocyte/macrophage population in SCLC is particularly associated with PLCG2‐high subpopulation [259]. TNF‐α‐producing macrophages can drive pancreatic cancer “classical” to “basal‐like” plasticity via reprogramming AP‐1enhancer [260].

In addition to TAMs, other factors within the tumor immune microenvironment can also drive cancer cell plasticity. Previous studies revealed myeloid‐derived suppressor cells could facilitate CSCs in ovarian, breast, and multiple myeloma [261, 262, 263]. Tumor‐associated neutrophils can promote liver and breast cancer stem‐like cells transmission [264, 265, 266]. Other researches focused on the inflammation factors. Kim and colleagues revealed IFN‐γ could induce melanoma cells revert to a more primitive cellular phenotype [267]. Raghavan and colleagues replicate “classical” to “basal‐like” lineage plasticity of pancreatic cancer in vitro via adding TGF‐β to the pancreatic cancer organoid [10].

Inflammation and tissue damage can lead to metaplasia and increases the risk of cancer [27]. This process appears to be similar to how the tumor immune microenvironment promotes cell plasticity, as discussed earlier. Indeed, recent studies using mouse models and large subject cohorts have elucidated the mechanisms of cell plasticity in tumor‐initiating inflammatory and tissue damage environments [20, 268, 269, 270]. In the lung cancer model, exposure to air pollutants triggers an influx of macrophages into the lung and the release of interleukin‐1β. This sequence of events induces a progenitor‐like cell state within EGFR‐mutant lung alveolar type II epithelial cells [269]. Tissue damage activation alarmin cytokine interleukin 33 facilitates acinar‐to‐neoplasia plasticity of the premalignant pancreatic epithelium [270].

Besides immune‐associate cells and factors, other components within the tumor microenvironment can also drive cancer cell plasticity. Miranda et al. demonstrate that mechanical signals in the tumor microenvironment, such as physical confinement, can trigger widespread chromatin remodeling in melanoma cells, inducing phenotypic plasticity [271]. Rapid tumor growth often outpaces the development of new blood vessels, leading to a hypoxic microenvironment [14]. HIFs can induce prostate cancer NE phenotype plasticity via FoxA2 and enable stemness of breast cancer through HIF‐2α‐SOD2‐mtROS‐PDI/GRP78‐UPR(ER) axis [272]. In hypoxic microenvironments, the stemness and differentiation capacity of CSCs are significantly heightened, primarily through the process of hypoxia‐induced EMT [273]. Additionally, high‐fat diet induced intestinal bile acids (BAs) dysregulation promotes Lgr5‐expressing CSCs adenoma‐to‐adenocarcinoma plasticity via BAs–farnesoid X receptor axis [274]. BAs can also promote EMT in cholangiocarcinoma through active CAFs in the microenvironment [275]. Moreover, in skin squamous cell carcinomas, stromal TGFβ‐mediated induction of leptin receptor (Lepr) and subsequent elevation of tissue leptin by the vasculature lead to enhanced LEPR–leptin signaling in CSCs. This signaling cascade activates the PI3K–AKT–mTOR pathway, driving the benign papilloma to massive squamous carcinoma transition [276].

In conclusion, we summarize cell‐intrinsic and ‐extrinsic enabling mechanisms that mediate cancer cell plasticity from the perspective of cancer hallmark. Actually, these factors often form a complex plasticity‐regulatory network. For example, Mutations of key enzymes of epigenetic regulation can lead to universal epigenome‐wide reprogramming and cancer cell plasticity [210, 277]. In addition, IL‐33, derived from the microenvironment, collaborates with KRAS mutations to activate epigenetic remodeling programs involved in early neoplasia and neoplastic transformation in pancreatic cancer [270]. The interconnected nature of these regulatory networks highlights the significant challenge of therapeutically controlling plasticity. Nevertheless, this same complexity also presents multiple opportunities to identify potential biomarkers for monitoring plastic cell states and therapeutic vulnerabilities. Therefore, translating these mechanistic insights into effective clinical strategies is a critical goal. Accordingly, the following section will discuss the progress and challenges associated with the clinical application of strategies designed to detect and target cellular plasticity.

## 4 Clinical Opportunities and Challenges of Cancer Cellular Plasticity

**FIGURE 4:**
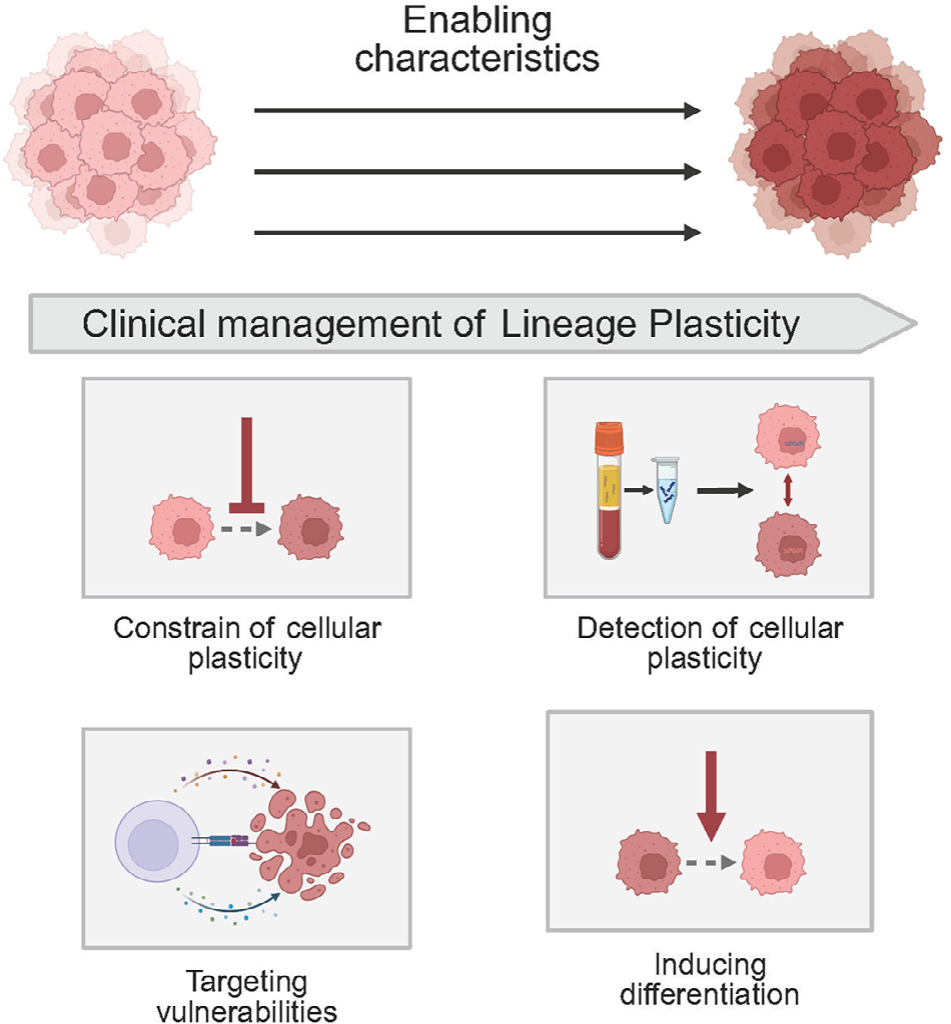
Clinical strategies for addressing lineage plasticity. Constraining cellular plasticity: Blocking the molecular pathways that enable a phenotypic change in cancer cells. Detection of cellular plasticity: leveraging convenient, noninvasive technologies for timely detection of cellular plasticity. Targeting the vulnerabilities of plastic cells: this approach identifies and exploits the unique weaknesses that emerge as cancer cells undergo a plastic transition. Inducing differentiation: this therapy forces immature, cancerous cells to differentiate into a mature, nondividing state, effectively neutralizing them.

Understanding the mechanisms that drive cellular plasticity is critical for translating this knowledge into clinical applications. Successfully targeting plasticity as a therapeutic vulnerability requires a dual approach. First, robust strategies must be developed to detect and monitor plastic cell states in patients. Second, therapeutic interventions are needed to either prevent these cellular transitions or eliminate the resulting malignant populations. This section discusses the progress, and challenges in both the clinical monitoring and therapeutic targeting of cancer cell plasticity (Figure 4).

### 4.1 Clinical Detect of Cellular Plasticity

Accurate pathological confirmation of a phenotypic switch is essential for guiding treatment in tumors exhibiting lineage plasticity. Clinically, this confirmation relies on tissue rebiopsy at the time of disease progression. This approach has several limitations, including its invasive procedure and the potential for sampling error due to intratumoral heterogeneity. Nevertheless, rebiopsy remains the gold standard for definitively diagnosing these plastic transitions [278, 279]. Clinically, lineage plasticity is identified when a biopsy of a recurrent or metastatic tumor reveals a histology—either mixed or fully converted—that is different from the primary diagnosis. Definitive confirmation of this phenotypic plasticity requires an integrated diagnostic approach. Genomic profiling establishes a shared clonal origin with the primary tumor (for example, by confirming a founder *EGFR* mutation) while also identifying secondary alterations that drive plasticity, such as the loss of *RB1* and *TP53* [25]. At the protein level, immunohistochemistry (IHC) validates lineage plasticity by demonstrating the loss of original lineage markers and the acquisition of markers characteristic of the new phenotype. The diagnostic utility of IHC for NE markers differs between tumor types. For instance, IHC has high sensitivity for detecting the small‐cell transformation of NSCLC, as these tumors consistently express at least one canonical NE marker (such as synaptophysin, chromogranin A, or CD56) [280]. In contrast, IHC is less reliable for transformed NE prostate cancer (T‐NEPC), as a subset of these tumors can be negative for this same marker panel [281]. Moreover, as an invasive procedure, biopsy poses risks to the patient and is impractical for the frequent, longitudinal sampling required to monitor a dynamic and often rapid biological process. The limitations of tissue‐based analysis necessitate the development of alternative, noninvasive strategies to monitor cellular plasticity.

Liquid biopsy is emerging as a powerful approach to detect cellular plasticity. By analyzing tumor‐derived material, such as circulating tumor DNA (ctDNA) and other analytes from a simple blood sample, liquid biopsy can provide a more comprehensive and dynamic portrait of the tumor's molecular landscape. This technique allows for safe, repeated sampling, enabling the longitudinal tracking of genomic and epigenomic alterations that drive and accompany plastic transitions [282]. Recently, several researches reveal that cell‐free DNA (cfDNA) holds significant promise for detecting both genetic and epigenetic changes associated with NE lineage plasticity in lung and prostate cancer. Talal et al. demonstrate that epigenomic cfDNA profiling can detect NE transformation in LUAD patients with EGFR mutant [282]. Recent studies have established that NEPC has a distinct cfDNA methylome compared with its parental adenocarcinoma, characterized by specific patterns: hypermethylation of ASXL3 and SPDEF and hypomethylation of NE drivers such as INSM1 [283, 284]. Targeted analysis of plasma cfDNA methylation offers a noninvasive method to detect NE plasticity, quantify tumor fraction, and classify diverse disease phenotypes using a composite scoring system [285]. Furthermore, Navonil et al. developed a prediction model based on ctDNA nucleosome positioning patterns associated with transcription regulation. This model achieved 97% accuracy for detecting dominant NE phenotypes and 87% accuracy for mixed NE phenotypes [286]. Recent technological advances in isolating circulating tumor cells (CTCs) now enable their direct, high‐fidelity transcriptomic profiling via RNA‐sequencing. This analysis has revealed that CTCs exist as distinct transcriptional subtypes that recapitulate the lineage heterogeneity of the primary tumor [287]. Collectively, these advancements show that a multianalyte liquid biopsy approach provides a powerful method to noninvasively monitor cellular plasticity.

Imaging technology is also emerging as a strategy to detect and monitor cellular plasticity. A first‐in‐human study has demonstrated the safety and feasibility of noninvasively detecting NE lung and prostate cancers using an immunoPET‐CT agent that targets the cell‐surface protein DLL3. In this trial, tumor uptake of the radiotracer—a zirconium‐89‐labeled anti‐DLL3 antibody ([⁸⁹Zr]Zr‐DFO‐SC16.56)—showed high concordance with DLL3 protein expression confirmed by IHC [288]. This study provides a potential noninvasive imaging‐based method for monitoring NE plasticity.

### 4.2 Pharmaceutical Interventions Targeting Lineage Plasticity

Therapeutic strategies to control cellular plasticity aim to intercept this process at three distinct stages. Therapeutic strategies to control cellular plasticity aim to intercept this process at three distinct stages. First, preventive approaches block the initial cellular plasticity procedure. Second, selective targeting exploits the unique vulnerabilities of established plastic cell states. A third approach, differentiation therapy, overcomes the maturation blocks that sustain certain plastic malignancies by forcing cancer cells into a more differentiated state that is either less aggressive or more therapeutically vulnerable. These clinical and preclinical strategies are summarized in Table 1.

**TABLE 1:** Therapeutic strategies targeting cellular plasticity in cancer.

	Target	Plasticity type	Cancer type	Interventions	Trial number	Status	References
**Prevent plasticity**	Chemotherapy	NE transdifferentiation	In patients with newly diagnosed lung adenocarcinoma with EGFR/TP53 RB1 alterations	Platinum, etoposide and Osimertinib	NCT03567642	Active, not recruiting	
	CDC7	NE transdifferentiation	Preclinical models of lung and prostate cancer with neuroendocrine plasticity	Simurosertib	NA	NA	[290]
	JAK/STAT	NE transdifferentiation and EMT	Preclinical models of neuroendocrine plasticity prostate cancer and patient derive organoid model	Ruxolitinib	NA	NA	[60]
	Netrin‐1	EMT	In patients with skin squamous cell carcinoma and preclinical models	NP137	NCT02977195	Completed	
	Snail and Twist	EMT	In patients with breast, colon, prostate, and pancreatic cancer		NCT05445791; NCT04559308; NCT04387630; NCT03359681; NCT02176161; NCT02614339; NCT03889795		
	HMG‐CoA	EMT	In patients with multitype cancer and preclinical models	Simvastatin	NCT05550415	recruiting	
	TGF‐β	EMT	In patients with resectable TNBC and preclinical models of bladder cancer, PDAC and ovarian cancer	in ref	in ref	in ref	[26, 299, 300]
**Target vulnerabilities**	Aurora kinase A	NE transdifferentiation	In patients with lung and prostate cancer with NE transdifferentiation	Alisertib, LY3295668	NCT04085315, NCT01799278	Recruiting, completed	[301, 302, 303, 304]
	Aurora kinase A/B	EMT	Preclinical models of EMT lung cancer	PF03814735	NA	NA	[106]
	PARP	NE transdifferentiation	Preclinical models of lung cancer with NE transdifferentiation	Olaparib+ durvalumab	NCT04538378	Terminated	[25]
	NOTCH	NE transdifferentiation	Preclinical models of lung cancer with NE transdifferentiation	Lurbinectedin	NA	NA	[305]
	DLL3	NE transdifferentiation	In patients with prostate cancer with NE transdifferentiation	Tarlatamab, Obrixtamig, MK6070	NCT04702737, NCT04471727, NCT04429087		[306, 307, 308, 309]
**Differentiation therapy**	PML‐RARy	NA	Acute promyelocytic leukemia (APL)	All‐trans retinoic acid (ATRA) and arsenic trioxide (ATO)	Standard of care [312]	NA	[312]
	LSD1 and WNT	NA	Preclinical model of acute myeloid leukemia (AML)	GSK–LSD1+ LY2090314	NA	NA	[313]
	Chemotherapy	EMT	In patients with metastatic breast cancer	Erbulin	NCT01669252, NCT00337103	Completed	[314, 315]
	HNF4α	NA	In patients with hepatocellular carcinoma	HNF4α srRNA‐LNP (CD‐801)	ChiCTR2300073093	Completed	[317]
	EZH2	NE transdifferentiation	In patients with prostate cancer with NE transdifferentiation	Tazemetostat	NA	NA	[318, 319]
	TET	NE transdifferentiation	Preclinical model of prostate cancer with neuroendocrine plasticity	Bobcat339	NA	NA	[58]
	MEK + PPARE	EMT	Preclinical model of breast cancer	Rosiglitazone + trematinib	NA	NA	[320]
	Cathesin H	Semisquamatization plasticity	Preclinical model of muscle‐invasive bladder cancers	E64	NA	NA	[72]

Abbreviations: EMT, epithelial–mesenchymal transdifferentiation; NE transdifferentiation, neuroendocrine transdifferentiation; TGF‐β, transforming growth factor‐beta.

The clinical trials data sources: https://clinicaltrials.gov/.

#### 4.2.1 Therapeutic Strategies to Constrain Lineage Plasticity

Based on the growing understanding of the molecular mechanisms that drive cellular plasticity, a primary therapeutic strategy involves intervening early to prevent the initiation of cellular plasticity.

As previously discussed, the concurrent loss of the tumor suppressors TP53 and RB1 is a key early event that initiates NE lineage plasticity in both lung and prostate cancer. To eradicate pre‐existing small‐cell clones and prevent NE lineage plasticity, Monica et al. administered a combination of platinum and etoposide—a small‐cell‐directed chemotherapy regimen—and the EGFR TKI Osimertinib to patients with EGFR/TP53/RB1‐mutant lung adenocarcinoma in a clinical trial [289]. This study represents an innovative clinical effort to proactively inhibit lineage plasticity. However, 5 of 11 patients still underwent small‐cell transformation, a result that highlights the need for a deeper mechanistic understanding to develop more effective preventive strategies. Furthermore, Upregulation of the cell cycle kinase CDC7 has been identified as a key early event in the NE transdifferentiation of lung and prostate cancers that harbor concurrent TP53 and RB1 mutations [290]. Preclinical in vivo studies have demonstrated that CDC7 inhibitor simurosertib can suppress the lineage plasticity and extend the efficacy of targeted therapies [290]. JAK/STAT and FGFR signaling is also a potential target to prevent NE lineage plasticity in prostate cancer [60, 165]. Preclinical studies using both murine and patient‐derived organoid (PDO) models have identified JAK/STAT pathway activation as a key event that initiates a state of high cellular plasticity. This plastic state is considered a prerequisite for subsequent, more stable lineage conversions, such as EMT or NE differentiation [60]. Pharmacological inhibition of JAK signaling both prevents the acquisition of plasticity and promotes reversion to a more differentiated, luminal epithelial state [60].

Targeting EMT and the associated process of EMP—key drivers of therapeutic resistance and metastasis in numerous malignancies—represents a critical clinical opportunity [26]. Two recent studies have demonstrated that netrin‐1 overexpression leads to EMT in mouse models. Furthermore, treatment with NP137, a netrin‐1‐blocking monoclonal antibody, has been shown to repress EMT in both preclinical models and a clinical trial [291, 292]. Metformin has been demonstrated to repress EMT and inhibit metastasis and therapy resistance in several preclinical studies [293, 294, 295]. Based on these, numerous clinical trials, including Phase 3 studies (e.g., NCT05445791), have evaluated metformin in various tumor types. However, these trials have so far failed to demonstrate a clear clinical benefit, such as EMT inhibition or improved patient survival [296, 297, 298]. This outcome is a cautionary reminder that preclinical efficacy does not always predict clinical benefit, underscoring the importance of robust data from patient trials. In addition, substantial preclinical evidence suggests that EMT can be suppressed through various interventions. These strategies include using repurposed compounds (such as omeprazole and simvastatin) or directly inhibiting key drivers like TGFβ signaling, NOTCH signaling, and core EMT‐TFs. However, the therapeutic potential of these approaches is limited by a lack of validating clinical data. For a more detailed discussion of these strategies to prevent the EMT and the mechanisms that govern it, readers are referred to several comprehensive reviews on this topic [26, 299, 300].

#### 4.2.2 Vulnerabilities to Target Lineage Plasticity

Although the optimal therapeutic strategy is to early detect and prevent cellular plasticity, the clinical reality is that these transitions are often detected only after a new phenotype is established. Therefore, an alternative therapeutic strategy is to target the specific vulnerabilities of these transformed cells. Based on a detailed understanding of the governing cell‐intrinsic and ‐extrinsic mechanisms, several strategies have been developed to specifically target the acquired dependencies of these plastic cell populations.

Aurora kinase A (AURKA) is a therapeutic target in NE‐transformed tumors, as its inhibition both degrades the oncogenic driver N‐Myc [301] and is synthetically lethal with RB tumor suppressor loss [302]. This dual rationale has prompted clinical trials of AURKA inhibitors in patients with resistant lung and prostate cancer [303, 304]. In EGFR TKI‐resistant lung cancer, inhibition of Aurora B kinase can inactivate the ATR–CHK1–Aurora B axis and induce apoptosis of EMT cells [106]. Building on preclinical evidence that PARP inhibition reduces SCLC growth and may enhance immunotherapy, a Phase II clinical trial is now assessing the combination of the PARP inhibitor olaparib and the checkpoint inhibitor durvalumab in patients with NE‐transformed tumors [25]. The dependency on Notch pathway suppression during the NE fate switch creates a key therapeutic vulnerability. Lurbinectedin exerts antitumor activity in NE‐transformed lung cancer by modulating NOTCH signaling [305]. As previously mentioned, Delta‐like ligand 3 (DLL3) is an atypical, inhibitory Notch ligand that is highly expressed on the surface of NE tumors. Moreover, the high surface expression of the inhibitory Notch ligand DLL3 on NE tumors makes it both a promising biomarker and an attractive therapeutic target. Building on this rationale, T‐cell engagers designed to target DLL3 have now been evaluated in both preclinical and early clinical studies [306, 307, 308, 309]. These therapeutic agents are designed to physically bridge a patient's T cells to the DLL3 protein on tumor cells, thereby inducing targeted cell killing. These initial studies have indicated promising antitumor activity, providing a strong rationale for the continued clinical development of this therapeutic strategy.

#### 4.2.3 Differentiation Therapy

Differentiation therapy, aims to reverse cellular plasticity itself, an approach particularly relevant for malignancies composed of highly plastic, undifferentiated cells. The goal is to use agents that induce a “redifferentiation” program, forcing malignant cells into a more stable and mature phenotype. This can diminish their aggressive potential and, in some cases, restore sensitivity to other therapies.

The classic example of this therapeutic concept is the use of all‐trans retinoic acid (ATRA) and arsenic trioxide (ATO) in acute promyelocytic leukemia (APL) [310]. These agents cooperatively target the APL‐defining PML‐RARα oncoprotein, which reactivates the suppressed transcriptional program for myeloid differentiation while also inducing apoptosis. This combination regimen is now the standard of care (SOC), transforming APL from a highly aggressive disease into a largely curable one [311, 312]. A study by Amir et al. showed that the simultaneous inhibition of LSD1 and GSK3 can suppress stemness and promote differentiation in a mouse model of acute myeloid leukemia (AML) [313]. Furthermore, recent studies demonstrate that the microtubule inhibitor eribulin can reverse EMT in breast cancer cells [314]. Analysis of paired biospecimens from patients treated with neoadjuvant eribulin suggests that the agent can induce a phenotypic switch from a basal‐like to a less aggressive, normal‐like subtype [315]. Forcing the re‐expression of the TF HNF4α, a master regulator of hepatocyte identity, is a promising differentiation‐based therapy for HCC [316]. Chuan and colleagues develop a novel platform that employs lipid nanoparticles (LNPs) to deliver an HNF4α‐encoding self‐replicating RNA (srRNA), providing sustained protein expression in HCC cells for up to 1 week [317]. Initial clinical studies have established that this therapy has a manageable safety profile and demonstrated preliminary evidence of efficacy [317].

Preclinical studies have also identified several other targets whose modulation can induce therapeutic differentiation. EZH2 inhibitor tazemetostat suppresses NE cell state and induce forward differentiation by downregulating key lineage‐driving TFs, such as ASCL1, and decommissioning the broader neuronal gene program [318]. In basal‐like TNBC, the concurrent inhibition of AKT and EZH2 first drives differentiation to a luminal phenotype and subsequently triggers apoptosis by co‐opting mammary gland involution pathways [319]. Targeting the epigenetic regulator TET2 represents another strategy to reverse NE plasticity and overcome resistance to AR‐targeted therapy [58]. Moreover, synergistic treatment with the MEK inhibitor trametinib and the PPARγ agonist rosiglitazone forces EMT‐derived aggressive breast cancer cells to transdifferentiate into benign and functional adipocytes, thereby extinguishing their oncogenic potential [320]. Inhibiting cathepsin H (CTSH) is a novel strategy to eliminate chemoresistant MIB with partial‐squamous plasticity. This approach works by simultaneously driving the tumor cells toward terminal squamous differentiation and triggering pyroptotic cell death [72].

## 5 Probing Cell Plasticity With Spatiotemporal Technologies

The significance of cancer cell plasticity in cancer evolution demands a detailed exploration of the molecular mediators and regulators driving this process. However, the dynamic and transient nature of cancer cell plasticity presents a formidable challenge for mechanistic studies. This process is not a simple switch but a continuous evolution of cellular states, deeply influenced by spatial context and temporal progression. Traditional research methods, which often provide only a static snapshot of bulk cell populations, have been unable to capture these intricate dynamics, leaving the specific regulators and intermediate states of plasticity poorly understood.

Recent tears, the revolution of spatiotemporal omics represents a significant approach for investigating the dynamic nature of cancer cell plasticity [321]. By integrating the capacity to resolve cellular evolutionary trajectories with the preservation of their in situ architectural context, these methodologies provide a powerful framework for mechanistic investigation. This enables researchers to deconstruct intratumoral heterogeneity, reconstruct cell state trajectories, and identify the specific microenvironmental factors that drive plastic transitions. In the following sections, we will detail the recent breakthroughs in spatiotemporal omics, focusing on their emerging applications in cell plasticity research and the significant implications these technologies hold for future investigations [321].

### 5.1 Lineage‐Tracing Technologies

**FIGURE 5:**
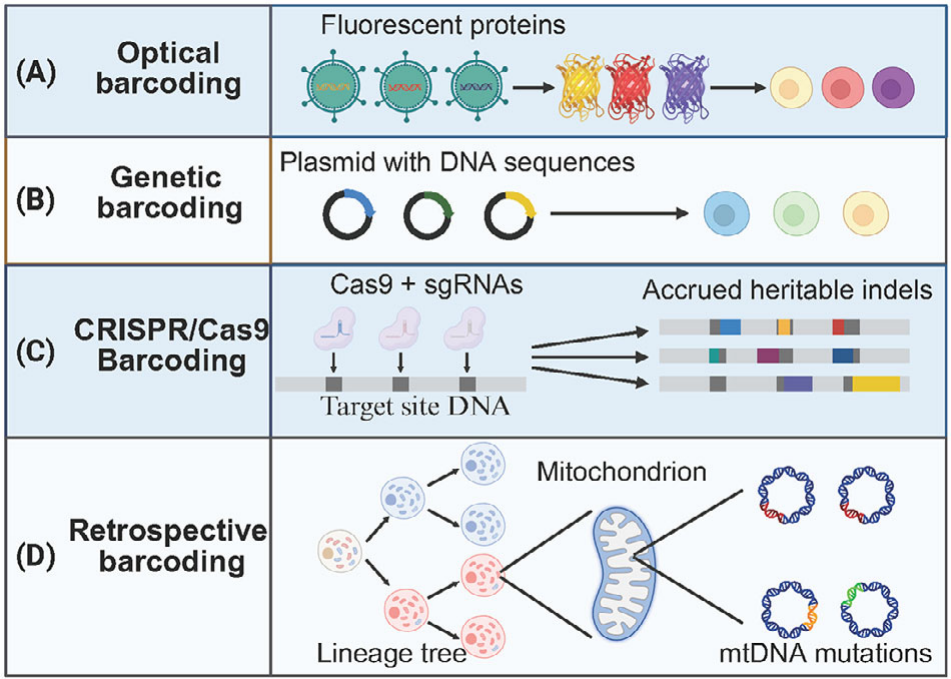
Four examples of lineage‐tracing technologies. (A) Cells incorporate specific fluorescent proteins via viral transduction, serving as barcodes that can be identified through optical methods. (B) In the genetic barcoding model, a unique nucleic acid sequence is integrated into the genome, enabling the descendants of marked cells to be tracked using sequencing techniques. (C) In the CRISPR/Cas9 barcoding model, cells are marked by accumulation of random errors, insertions and deletions during CRISPR/Cas9 editing of integrated genomic target sites. (D) In the retrospective barcoding model, mitochondrial DNA mutations are used as natural barcodes to trace lineage and generate phylogenetic relationships between different cells. mtDNA, mitochondrial DNA; sgRNAs, single guide RNA.

Cell plasticity is a dynamic and continuous biological process. Much of the previous research has primarily focused on observing the beginning and end points of these transitions. Actually, the intermediate states contain crucial information essential for understanding cell plasticity and tumor evolution. The hybrid EMT state discussed above serves as a suitable example. Indeed, recent developments in single‐cell transcriptomics provide a comprehensive method for capturing cellular plasticity dynamics through collecting and sequencing cells at different stages. With these data, computational algorithms can reconstruct cell trajectories along a proposed hierarchy [322]. Previous studies have employed this system to elucidate the temporal evolution of both ARPC transdifferentiation [57] and intratumor heterogeneity in SCLC [323]. However, this strategy cannot directly capture the long‐term dynamic relationships between individual cells. To establish cell states across different time points, lineage tracing is considered the most reliable technique (Figure 5).

The optical barcoding strategy using fluorescent proteins is a classical lineage‐tracing technology in cell plasticity research. For example, Zou et al., using YFP‐labeled prostate luminal cells, demonstrated that AR‐resistant NE cells emerge through the transdifferentiation of luminal adenocarcinoma cells, rather than originating from NE or basal cells [324]. Moreover, a in situ fluorescent proteins lineage‐tracing study revealed that PIK3CA(H1047R) can induce CSC plasticity and intratumor heterogeneity in the breast cancer mouse model [195]. Moreover, the optical barcoding model with dual recombinase has been used to illustrate how partial and complete EMT states contribute to metastasis [325, 326]. However, the range of fluorescent proteins limits the number of cell populations that can be traced.

Recent studies have reported genomic barcoding models, where each founder cell is marked with a unique nucleic acid sequence, typically delivered through viral vectors [327, 328, 329]. This sequence integrates into the genome and is inherited by all subsequent generations, enabling the identification and tracking of entire clones throughout their proliferation. The diversity of barcodes is directly determined by the number of founder cells initially labeled. This model is also used in tumor cell plasticity studies. In their study on cellular plasticity across the four primary cellular states in glioblastoma, Neftel and colleagues combined scRNA‐seq with genomic barcoding in both a genetic mouse model and PDX models to demonstrate cellular transitions at single‐cell resolution [181]. Indeed, Gutierrez et al. developed a genomic barcoding system, ClonMapper [330]. In this elegant system, random sgRNA sequences are used to generate barcodes. When a Cas9 transcriptional activator with a specific barcode‐binding sequence is introduced, synthetic gene circuits are activated, resulting in clone‐specific fluorescent reporter expression. It integrates DNA barcoding with scRNA‐seq and clonal isolation, offering a novel approach to cancer cell plasticity research. However, genomic barcoding systems require ex vivo manipulation of cells, which limits their application.

The advancement of flexibly inducible Cas9‐enabled lineage‐tracing techniques offers a broader scale and finer resolution compared to earlier methods, enabling deeper exploration of cellular dynamics and evolutionary trajectories [331]. Briefly, Cas9 target sequences and their corresponding guide RNAs are introduced into the host genome [332]. Upon activation of the inducible Cas9 system, diverse DNA mutations are generated within a defined locus through genome editing. These mutational outcomes are transcribed, allowing for lineage tracing at the single‐cell level [332]. Applying this approach, Bowling and colleagues developed the CRISPR array repair lineage tracing (CARLIN) mouse line, enabling lineage‐tracing barcodes to be induced at any stage of development in vivo [333]. Weissman laboratory has used lineage‐tracing models based on Cas9 to explore metastasis process in a lung cancer xenograft mouse model [334]. In addition, they developed a brilliant in situ lung cancer mouse model with Cas9‐enabled lineage tracing [335]. This system allows for tracking tumor evolution from single transformed cells to metastatic tumors with unparalleled resolution [335]. By employing this lineage‐tracing approach, their study provides crucial insights into the role of cell state plasticity in various stages of tumor evolution, offering a valuable tool for understanding tumor dynamics. Indeed, Li et al. recently reported a DARLIN mouse model, which features an expansive lineage‐barcode capacity and high‐fidelity lineage recovery within single‐cell assays. Uniquely, this platform facilitates the concurrent multiomic profiling of DNA methylation, chromatin accessibility, and gene expression, alongside the resolution of lineage information at the single‐cell level.

The techniques mentioned above require advanced exogenous barcode sequences and are not suitable for human studies, limiting their application in research involving humans. A retrospective approach employs strategies that analyze a population of cells, reconstructing their lineage by performing phylogenetic analysis based on shared genetic variants. Ludwig et al. reported that somatic mutations in mitochondrial DNA (mtDNA) can be tracked by single‐cell assay for transposase accessible chromatin (scATAC) sequencing [336]. These mtDNA mutations serve as natural, unique barcodes, offering valuable insights into cellular lineage and dynamics [336]. In colorectal cancer, single‐cell mtDNA sequencing can be utilized as clonal markers, enabling the resolving of intratumor heterogeneity. We recommend a recent review that summarizes significant advances in mitochondrial genetics and highlights its potential applications in tracking clonal evolution and heterogeneity in human cancer specimens [337]. Additionally, Xiao et al. devised EpiTrace, a computational methodology designed to quantify cellular age and reconstruct lineage trajectories across diverse taxa and cell lineages through the utilization of scATAC‐seq data [338]. The operational basis of this platform exploits age‐dependent chromatin accessibility (ChrAcc) at clock‐like loci as an intrinsic molecular barcode, characterized by alterations that are concomitant with mitotic events and cell state transitions [338]. Taken together, the integration of high‐throughput single‐cell profiling techniques and diverse barcoding systems has provided unprecedented insights into the transitions of cancer cells through various states. These innovations establish a solid foundation for future research into the mechanisms of cell plasticity in tumor evolution.

### 5.2 Spatiotemporal Omics

The cancer cell‐extrinsic microenvironment plays a key role in cellular plasticity and tumor evolution. While the development of single‐cell sequencing technology has revolutionized the characterization of cellular heterogeneity, conventional dissociation‐based approaches are constrained by the loss of spatial context. Conversely, the emergence of spatial omics technologies has facilitated the high‐resolution mapping of cellular composition, topological organization, intercellular signaling, and the spatial dynamics of the cellular ecosystem [339]. Indeed, several previous studies investigating the relationship between cell plasticity and spatial organization within tumors have employed spatial omics technologies to reveal critical insights into how these factors influence tumor behavior and progression [133, 182, 256, 340, 341, 342]. Varrone et al. recently presented CellCharter, an innovative algorithmic framework that enables the identification, characterization, and comparison of cellular niches in spatially resolved datasets, offering new insights into the relationship between spatial tissue architectures and cell plasticity [343].

Cutting‐edge technologies for single‐cell analysis have evolved our ability to explore and understand cell plasticity, leading to important new insights. To fully elucidate the multidimensional mechanisms of cell plasticity and tumor evolution, it is essential to integrate molecular‐level sequencing information with insights into how individual cells operate within their complex networks of interactions, forming functional biological systems. The integration of spatiotemporal multiomics technologies, along with advancements in artificial intelligence (AI) and computational biology, represents a promising future direction for research. For a comprehensive understanding, we recommend consulting recent reviews that detail the methods and applications of these technologies [321, 322, 339, 344, 345].

## 6 Conclusions

Cancer cell plasticity is a hallmark of cancer. Previous research has increasingly revealed cellular plasticity as an indispensable intermediate process in tumor evolution. Specifically, it can be an immediate consequence of enabling characteristics such as genomic mutations, epigenetic reprogramming, transcriptional remodeling, and the abnormal microenvironment. Meanwhile, it directly facilitates other oncogenic features, including malignant transition, intratumor heterogeneity progression, therapy resistance, and distal metastasis. Moreover, the CSC cell state can also be integrated in the cellular plasticity. In this review, we emphasize the central role of cell plasticity in tumor evolution and summarize its interactions with other cancer hallmarks. This perspective offers new insights into understanding the complex and dynamic nature of tumor evolution through the lens of cell plasticity.

The complexity and dynamic nature of cellular plasticity pose a major clinical challenge, as this process contributes to therapeutic failure and disease recurrence. However, an improved understanding of the underlying mechanisms is now leading to new clinical opportunities in two main areas. First, noninvasive monitoring strategies, such as liquid biopsies, are being developed to track these cellular transitions in patients. Second, this mechanistic knowledge is guiding the development of novel therapies categorized into three approaches: preventing plastic transitions, targeting the vulnerabilities of plastic cells, and reversing plasticity through differentiation therapy. The translation of these insights into the clinic represents a fundamental shift from reacting to established resistance to proactively managing the processes that drive it.

Despite these advances, the molecular mediators that govern plasticity remain largely unknown due to the transient and context‐dependent nature of these cellular states. A primary goal of future research is therefore to capture these dynamics. Recent advances in spatiotemporal multiomics provide an opportunity to address this challenge. By integrating lineage‐tracing technologies, such as CRISPR‐based barcoding, with single‐cell and spatial omics, researchers can reconstruct cellular trajectories while preserving their spatial context in situ. This approach will enable the creation of detailed atlases of cancer cell plasticity to identify key intermediate states, their molecular drivers, and the microenvironmental niches that regulate these transitions.

Ultimately, a deeper, systems‐level understanding of cancer cell plasticity is essential for future advances in oncology. Harnessing these insights, enabled by new spatiotemporal technologies, will not only uncover fundamental mechanisms of cancer plasticity but also directly inform clinical practice. This knowledge is critical for developing more accurate methods for disease stratification, designing therapeutic strategies to anticipate or prevent resistance, and advancing the goal of precision medicine. The long‐term vision is to shift from a reactive therapeutic approach, which merely responds to emergent resistance, to a proactive strategy that manages the entire clinical course. This proactive approach includes predicting a tumor's plastic potential at diagnosis, monitoring cellular state transitions during therapy, and deploying precision interventions to guide its evolutionary trajectory toward manageable outcomes.

## Author Contributions

Writing the manuscript, Shangwei Sun, Yunwei Sun; preparing figures, Yunwei Sun, Ling Lan, Siyuan Luan; revising the manuscript, Jin Zhou, Jiehui Deng, Yong Yuan, Zhong Wu; conceptualization and supervision, Yong Yuan, Zhong Wu. All the authors read and approved the final manuscript.

## Funding

This study was partially supported by the National Natural Science Foundation of China (Grant Nos. 81970481, 82272685, 82273018), the National Science and Technology Major Project of China (2025ZD0552413), the Sichuan Science and Technology Program (2024NSFSC0734, 2025NSFJQ0063, and 2025ZNSFSC1912), 1.3.5 project for disciplines of excellence (2023SCUH0062) and Artificial Intelligence (ZYAI24049), West China Hospital, Sichuan University, and Chengdu Science and Technology Program (2024‐YF05‐00448‐SN).

## Ethics Statement

The authors have nothing to report.

## Conflicts of Interest

The authors declare no conflicts of interest.

## Data Availability

The authors have nothing to report.
